# Concerns Regarding Masataka et al.'s “Revisiting the Gateway Drug Hypothesis for Cannabis: A Secondary Analysis of a Nationwide Survey Among Community Users in Japan”

**DOI:** 10.1002/npr2.70057

**Published:** 2025-09-10

**Authors:** Zui C. Narita

**Affiliations:** ^1^ Department of Behavioral Medicine, National Institute of Mental Health National Center of Neurology and Psychiatry Kodaira Japan

**Keywords:** absolute risk, causal inference, odds ratio, prediction models, public health, research ethics, research integrity, scientific rigor, substance epidemiology, Table 2 Fallacy

## Abstract

Masataka et al.'s cannabis gateway study misrepresents the 43.8% probability of cannabis users transitioning to illegal drugs as “rare,” and misuses regression via the Table 2 Fallacy. These critical issues discredit their conclusion.

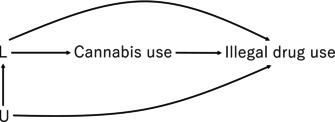

I read with interest the article “Revisiting the Gateway Drug Hypothesis for Cannabis: A Secondary Analysis of a Nationwide Survey Among Community Users in Japan” by Masataka et al. (2025) [1]. The authors conclude that their findings “challenge the gateway hypothesis in the Japanese context,” particularly emphasizing the odds of progression from cannabis to other illegal substances and the role of social determinants. While I appreciate the effort to describe substance use patterns in Japan, I would like to raise two critical issues regarding their inferential interpretation of the results.

First, the authors stated:

Even when considering the broader category of illegal drugs, including substances like MDMA, the odds remained below 1 (0.78), indicating that most cannabis users did not proceed to use other illegal substances. These findings challenge the notion that cannabis alone should be labeled a ‘gateway drug,’ as such a claim appears scientifically unsupported within the Japanese context.

They further concluded in the abstract that “Cannabis use in Japan typically follows alcohol and tobacco, and rarely leads to further drug use.”

However, the odds of 0.78, when converted into probability, correspond to 43.8%—indicating that nearly half of cannabis users did transition to other illegal substances after cannabis use. This probability is far from “rare,” and characterizing it as such constitutes both a misrepresentation of the data and a rhetorical spin that downplays the observed findings, thereby distorting science [2]. Moreover, drawing a meaningful conclusion would require comparison with non‐users—a critical element absent from the study design. Of course, this study also suffers from other major limitations—including its cross‐sectional design, selection bias, and measurement bias—rendering it inconclusive. Nonetheless, the authors misrepresent the implications of their findings in a way that misleads readers and directly contradicts their own data.

Second, the authors appear to have misused the prediction model in a way that reflects a more systematic flaw. They wrote:

Rather than implying a causal gateway effect of cannabis use, these results highlight the importance of considering the broader life context in which substance use occurs. Social determinants such as age cohort, educational background, and socioeconomic position appear to shape patterns of substance progression independently of the pharmacological properties of cannabis.

While acknowledging the importance of social determinants might sound reasonable at first glance, the conclusion suffers from a well‐known statistical pitfall referred to as the Table 2 Fallacy [3]. The authors used multivariable logistic regression, including variables such as age, education, employment status, and history of mental disorders. However, when variables are included without a clear causal framework (e.g., directed acyclic graphs) [4] to distinguish between exposures, confounders, mediators, and colliders, the interpretation of estimates becomes severely compromised. Inserting multiple variables into a model without considering their causal roles and making interpretations from the obtained estimates does not “highlight the importance”—rather, it distorts science. The misuse of prediction models in this way is unfortunately common [5], yet it is unjustifiable and should not persist in scientific practice.

Given these serious concerns—particularly the misrepresentation in presenting the probability of progression from cannabis to other illegal substance use, and the reliance on the Table 2 Fallacy—the study's conclusions require substantial re‐evaluation. The paper contains critical issues in its inferential claims, which should be avoided in scientific literature. This is especially true in the field of epidemiology, where research findings often have immediate implications for public policy. Once misinformation spreads, it cannot be fully retracted or undone. Researchers and reviewers must be vigilant in ensuring appropriate modeling practices and valid interpretations, especially when drawing conclusions that may shape public discourse and legislation.

## Ethics Statement

The author has nothing to report.

## Consent

The author has nothing to report.

## Conflicts of Interest

The author declares no conflicts of interest.

## Data Availability

The author has nothing to report.
